# The role of unintended pregnancy in internalized stigma among women living with HIV in Kenya

**DOI:** 10.1186/s12905-021-01224-5

**Published:** 2021-03-17

**Authors:** Sara Chace Dwyer, Aparna Jain, Wilson Liambila, Charlotte E. Warren

**Affiliations:** 1 Population Council, 4301 Connecticut Ave NW # 280, Washington, DC 20008 USA; 2 Population Council, Nairobi, Kenya

**Keywords:** Internalized stigma, Family planning, Unintended pregnancy, Reproductive health, HIV-related stigma and discrimination

## Abstract

**Background:**

Kenya has successfully expanded HIV treatment, but HIV-related stigma and discrimination, and unintended pregnancy remain issues for many Kenyan women living with HIV. While HIV-related stigma can influence the health seeking behaviors of those living with HIV, less is known about how reproductive health outcomes influence internalized stigma among women living with HIV.

**Methods:**

Baseline data only were used in this analysis and came from an implementation science study conducted in Kenya from 2015 to 2017. The analytic sample was limited to 1116 women who are living with HIV, between 18 to 44 years old, and have ever experienced a pregnancy. The outcome variable was constructed from 7 internalized stigma statements and agreement with at least 3 statements was categorized as medium/high levels of internalized stigma. Unintended pregnancy, categorized as unintended if the last pregnancy was mistimed or unwanted, was the key independent variable. Univariate and multivariate logistic regression models were used to assess the association between unintended pregnancy and internalized stigma. Associations between internalized stigma and HIV-related discrimination and violence/abuse were also explored.

**Results:**

About 48% agreed with at least one internalized stigma statement and 19% agreed with at least three. Over half of women reported that their last pregnancy was unintended (59%). Within the year preceding the survey, 52% reported experiencing discrimination and 41% reported experiencing violence or abuse due to their HIV status. Women whose last pregnancy was unintended were 1.6 times (95% CI 1.2–2.3) more likely to have medium/high levels of internalized stigma compared to those whose pregnancy was wanted at the time, adjusting for respondents’ characteristics, experiences of discrimination, and experiences of violence and abuse. Women who experienced HIV-related discrimination in the past 12 months were 1.8 times (95% CI 1.3–2.6) more likely to have medium/high levels of internalized stigma compared to those who experienced no discrimination.

**Conclusions:**

Results suggest that unintended pregnancy is associated with internalized stigma. Integrated HIV and FP programs in Kenya should continue to address stigma and discrimination while increasing access to comprehensive voluntary family planning services for women living with HIV.

**Supplementary Information:**

The online version contains supplementary material available at 10.1186/s12905-021-01224-5.

## Background

In Kenya, it is estimated that 1.6 million people (approximately 3% of Kenya’s population) are living with HIV as of 2018 and women ages 15 years and older account for about 57% of all individuals living with HIV [1]. This is slightly higher than the global average, where women account for 50% of those living with HIV [2]. The HIV prevalence rate for women of reproductive age (15–49 years) in Kenya is almost double the rate for men of the same age (6.1 compared to 3.4, respectively) [1].

Kenya has successfully expanded access to HIV services in the last decade. Among Kenyan women living with HIV (WLHIV), about 75% are on antiretroviral therapy (ART) in 2018 compared to 29% in 2010 [1]. Most pregnant WLHIV (91%) in Kenya receive antiretroviral drugs as part of prevention of mother-to-child transmission strategies [1], which has been proven to significantly reduce the risk of transmission [3]. Prevention of mother-to-child transmission strategies have led to an estimated 1.4 child infections averted [4] making it possible for women and couples living with HIV to have children without transmitting HIV during pregnancy and the postpartum period [5]. Yet WLHIV who wish to avoid pregnancy also need comprehensive family planning (FP) services [6]. Studies in Kenya have shown that WLHIV want to avoid pregnancy more so compared to HIV-negative women [6–8], consistent with other studies conducted in Malawi [7, 9], sub-Saharan Africa broadly [10], and the United States [11]. Women’s fertility decisions have been found to change within one week of receiving a HIV-positive diagnosis [9] and reasons for wanting fewer children include concerns over transmitting HIV to their child, not being able to care for their children, greater risk to the mother’s health, and increased financial burden [7, 8, 11]. Some studies have found that individuals living with HIV may decide to have additional children as they begin to feel healthier with ARTs [10, 12].

While many HIV-positive women may want to avoid a pregnancy, unintended pregnancy (the percentage of pregnancies that were wanted later or not wanted at all) is generally high among WLHIV. In Kenya, unintended pregnancy among WLHIV was found to be between 43 and 59% [6, 8, 13], which is higher than the national estimates for all women age 15–49 (36%) [14]. Studies conducted in other countries have also found high levels of unintended pregnancy among WLHIV: between 68 and 71% in South Africa [15–17]; 66–92% across sub-Saharan Africa [10]; 60% in Canada [18]; and 83% in the United States [19]. Evidence of differences in unintended pregnancy comparing WLHIV and HIV-negative women is mixed. Studies in Kenya [6], Ethiopia [20] and South Africa [21] found WLHIV reported higher rates of unintended pregnancy compared to HIV-negative women. A study conducted in Nigeria and Zambia, however, found no difference in the odds of unintended pregnancy by HIV status [22]. The evidence around contraceptive use among WLHIV compared HIV-negative Kenyan women is also mixed [6, 7, 23].

### HIV-related stigma and discrimination

HIV-related stigma and discrimination involve negative attitudes and behaviors anyone can have, including family, friends, health care workers, and community members, towards people living with HIV [24]. People living with HIV can also experience internalized stigma, by having negative feelings and thoughts about themselves because of their HIV status [25], and anticipated stigma, which is the belief that they will experience stigma, discrimination or abuse from others because of their HIV status [26]. HIV-related stigma and anticipated stigma can influence the health seeking behaviors of people living with HIV including ART adherence [16, 27–32]. A systematic review conducted in 2013 found mixed results on the effects of internalized stigma on ART adherence [28] but more recent studies have documented an association between internalized stigma and low ART adherence [32–34] and low ART initiation [35]. In Kenya, anticipated stigma was found to influence HIV testing among pregnant women [26, 31] while HIV-related stigma and discrimination were reasons why pregnant WLHIV reported avoiding delivery in a health facility [29]. Internalized stigma can also influence a person’s wellbeing and has been linked to depression and thoughts of suicide. For instance, postpartum women in Kenya who experienced shame over their HIV status were more depressed compared to postpartum women who reported social rejection [36]. Qualitative studies in Kenya have also shown that some WLHIV prefer to receive FP services from facilities specializing in HIV services [30].

The effects of HIV-related stigma and discrimination on women’s wellbeing and health-seeking behaviors have been well documented. Little is known, however, about the association between reproductive health outcomes and internalized stigma experienced by WLHIV. This paper aims to contribute to the literature by examining the relationship between unintended pregnancy and internalized stigma among HIV-positive women in south western Kenya.

### Study locations

The study was conducted in Samia and Butula sub-counties of Busia County, which are located in south western Kenya. Samia has a population of 107,176 and Butula has a population of 140,334 [37]. Both sub-counties have similar socioeconomic characteristics [38]. The HIV prevalence in Busia county is 10%, double the national average (5%) for adults aged 15–64 years [39]. The estimated HIV prevalence rate among women is 9% in Busia county compared to 5% nationally [40].

## Methods

### Data source

The baseline data used in this analysis were part of larger implementation science study that tested a community-based approach for integrated FP and HIV services between 2015 and 2017. Quantitative interviews were conducted with WLHIV before and after the community-based FP/HIV intervention was implemented [38]. The intervention did not look at reducing HIV-related stigma. The study protocol received IRB approvals from the Population Council (Protocol 702) and the Kenyatta National Hospital/University of Nairobi Ethics Research Committee (Protocol P573/08/2015).

Women between the ages of 18–49 were enrolled through HIV support groups. The support groups provided their members with information about the study and women who were interested in participating were contacted by research assistants trained in research ethics, implementing informed consent forms, and the study’s design, objectives, and questionnaires. The research assistants then met with potential respondents, provided them with the purpose and objectives of the study and obtained informed consent. Research assistants used tablets to administer a quantitative questionnaire. The questionnaire collected data on socio-demographic characteristics, general health status, health-seeking behaviors, FP use, use of HIV services, and perceived or experienced stigma and discrimination (Additional file 1). Interviews were conducted in Kiswahili and responses in the tablet were recorded in English. A total of 1609 women participated in the implementation science study: 1090 in Samia and 519 in Butula.

### Dependent variable

The dependent variable is internalized stigma, categorized as medium/high versus low. The internalized stigma variable was created from seven questions that were adapted from the HIV Stigma Scale [41]. Respondents were asked to answer a series of attitudinal questions on a 4-point Likert scale ranging from strongly disagree to strongly agree. Responses for each item were then dichotomized into strongly agree/agree or strongly disagree/disagree. The seven items were combined to form an additive index ranging from 0 to 7, where higher values represented higher levels of internalized stigma. The internalized stigma variable was dichotomized into low and medium/high internalized stigma. Low internalized stigma included women who agreed with 0 to 2 items (coded as 0) and medium/high internalized stigma included those who agreed with 3 or more items (coded as 1).

### Independent variables

#### Unintended pregnancy

Women were asked “At the time you became pregnant with your last child, did you intend to become pregnant then, did you want to wait until later, or did you not want to have any (more) children?”. A dichotomized variable was created where unintended pregnancy included women who reported that their last pregnancy was wanted later or not at all (coded as 1) and intended pregnancy included women whose last pregnancy was wanted at the time they became pregnant (coded as 0).

#### Experience of HIV-related discrimination and violence or abuse

Two variables, experience of HIV related discrimination and experience of violence or abuse, were used to adjust the model because of their known relationship to internalized stigma. Women were asked about their experiences with discrimination related to their HIV status. They were asked four questions about discrimination that occurred in the past 12 months: excluded from family activities; aware of being gossiped about; rejected by a sexual partner; and treated unfairly or discriminated against by a healthcare provider. Women who did not experience discrimination related to any of the four questions were coded as 0, and women who experienced 1 or more of the questions were coded as 1-

Experience of violence or abuse was measured by three questions. Women were asked if they were verbally insulted, physically abused, and sexually assaulted or forced to have sex in the past 12 months. A dichotomized variable was constructed where 1 was coded if the respondent had answered yes to at least one questions and 0 if she did not experience violence on all 3 questions.

#### Additional variables

Additional covariates included age, marital status, number of living children, having one or more children living with HIV, education, length of time living in same village, self-reported health status, length of time using antiretrovirals (ARVs) and sub-county residence.

### Data analysis

The analytic sample was limited to respondents who were 18 to 44 years of age and had ever been pregnant or were currently pregnant (n = 1116) at the time of the survey. Descriptive statistics were calculated for respondent characteristics, unintended last pregnancy, HIV-related discrimination, experience of violence, and reported experience of internalized stigma. Univariate and multivariate logistic regression models were used to assess the association between unintended pregnancy and internalized stigma. The analysis were conducted in STATA.SE, Version 13.

## Results

Table 1 shows the demographic profile of respondents living with HIV, and experience of internalized stigma, HIV-related discrimination, and violence or abuse. Over half (56%) of women were in the latter half of their reproductive life (30% were aged 35–39 and 26% were aged 40–44). Many (63%) were married at the time of the interview and 42% had between 3–4 living children. Few women (13%) reported that one or more of their children were HIV positive. Most women did not complete primary school (78%), and 68% lived in the same village for over 10 years. More women were from Samia sub-county (70%). The majority rated their health as fair or good (90%) and 78% were using ARVs for over a 1 year.

**Table 1:** Study sample characteristics (n = 1116)

	%
Age	
18–24	6.7
25–29	16.6
30–34	20.8
35–39	29.9
40–44	26.0
Marital status	
Single	2.6
Living together	2.7
Married	62.8
Divorced/separated/widowed	31.9
Number of living children	
0	2.5
1–2	23.8
3–4	41.6
5+	32.1
Number of children living with HIV	
At least 1	12.5
None/don’t know	87.5
Education	
None	9.9
Did not complete primary	78.3
Completed primary	10.8
Secondary or more	1.0
Duration living in same village	
< 10 years	32.0
≥ 10 years	68.0
Sub-County	
Samia	70.3
Butula	29.7
Self-reported health status	
Poor/Don’t know	9.5
Fair	41.3
Good	49.2
Length of time on ARV treatment	
No treatment/1 year or less	21.9
More than 1 year	78.1
Unintended last pregnancy	
Yes (wanted pregnancy later/did not want pregnancy at all)	58.6
No (wanted pregnancy then)	41.4
Internalized stigma	
Medium/high	19.1
Low/none	80.9

More than half (59%) of women reported that their last pregnancy was unintended (Table 1). Among those who reported that their last pregnancy was unintended (n = 654), 57% were not using contraception at the time and the most commonly cited reasons for not using FP among these women included: didn’t know about FP or where to get it/lack of access (14%); partner didn’t want them to use FP or others advised them against using FP (14%); fear of side effects/because contraceptive methods interfered with their bodily processes (12%); and not wanting to use FP (10%) (data not shown). Nineteen percent of women experienced medium/high level of internalized stigma (Table 1).

**Fig. 1:**
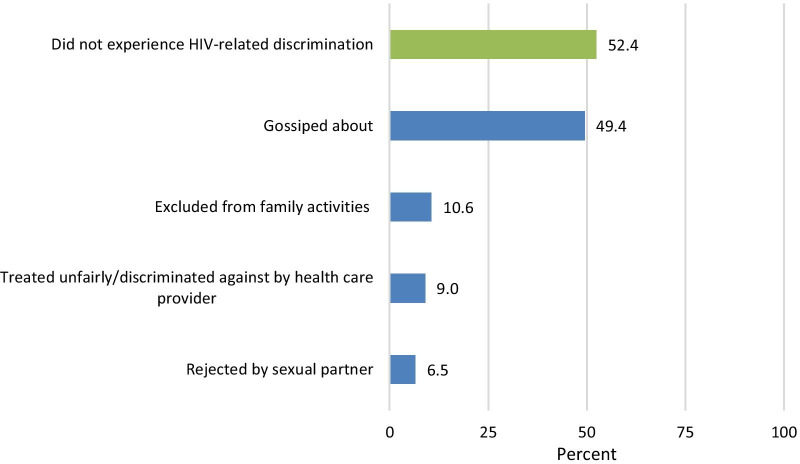
Percent of respondents who experienced HIV-related discrimination in the 12 months preceding the interview (n = 1116)

Figure 1 shows the percent of respondents who experienced HIV-related discrimination in the 12 months preceding the interview. Just over half of respondents did not experience any HIV related discrimination. Just under half said that they were gossiped about because of their HIV status and 11% were excluded from family activities. Few said that they were treated unfairly by a health care provider (9%) or were rejected by a sexual partner (7%).

**Fig. 2:**
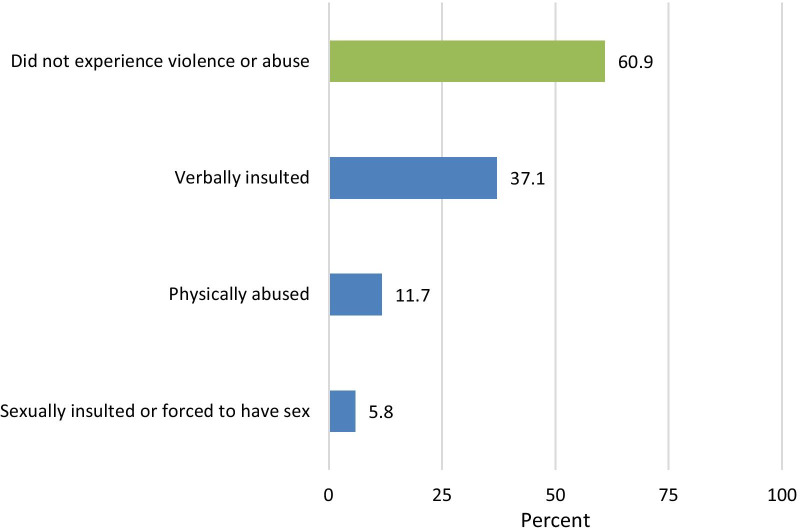
Percent of respondents who experienced violence or abuse in the 12 months preceding the interview (n = 1116)

Figure 2 shows the percent of respondents who experienced violence within the 12 months preceding the interview. Thirty-seven percent had been verbally insulted and 12% had been physically abused. Six percent had been sexually insulted or forced to have sex within the past 12 months.

### Internalized stigma

**Fig. 3:**
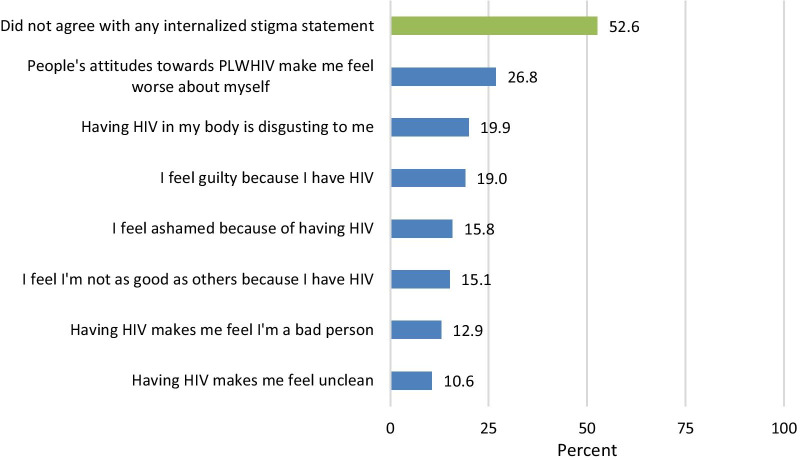
Percent of respondents who agreed with internalized stigma statements (n = 1116)

**Fig. 4:**
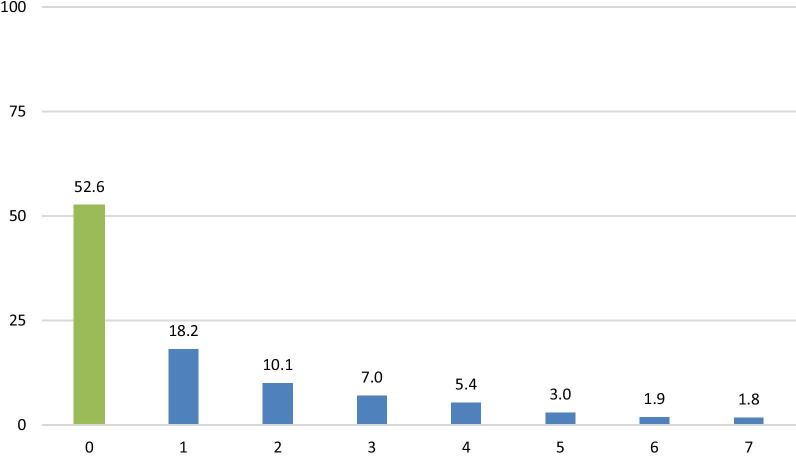
Distribution of respondents by number of internalized stigma statements agreed to (n = 116)

Figure 3 shows women’s responses to seven internalized stigma statements. Over a quarter (27%) of women agreed with the statement “people’s attitudes towards people living with HIV makes me feel worse about myself.” Twenty percent agreed with the statement “having HIV in body is disgusting to me” and 19% agreed with “I feel guilty because I have HIV.” Between 11 and 16% agreed with the other four internalized stigma measures. Figure 4 shows the percent of respondents who agreed with multiple internalized stigma statements. More than half (53%) did not agree with any internalized stigma statement, 18% agreed with 1 statement, 10% with 2 and 19% with 3 or more. Only 2% agreed with all 7 statements.

Unadjusted and adjusted odds ratios of medium/high internalized stigma are presented in Table 2. Women who reported that their last pregnancy was unintended were 1.7 times more likely to have higher levels of internalized stigma compared to those who reported that their last pregnancy was intended (OR 1.7; 95% CI 1.3–2.4). When adjusting for experience of discrimination, experience of violence or abuse and other respondent characteristics, the effect of unintended pregnancy reduced slightly to 1.6 but remained statistically significant (AOR 1.6; 95% CI 1.2–2.3).

**Table 2:** Unadjusted and adjusted logistic regression models of unintended last pregnancy on medium to high internalized stigma among women who have ever been pregnant (n = 1116)

	Unadjusted Model		Adjusted model	
	OR	95% CI	AOR	95% CI
Unintended last pregnancy				
Yes	1.74***	(1.26–2.39)	1.64**	(1.16–2.30)
No	Ref		Ref	
Experienced HIV-related discrimination				
Yes	2.02***	(1.48–2.77)	1.79***	(1.25–2.58)
No	Ref		Ref	
Experienced HIV-related violence/abuse				
Yes	1.63***	(1.21–2.20)	1.17	(0.82–1.66)
No	Ref		Ref	
Length of time on ARV treatment				
No treatment/1 year or less	0.83	(0.58–1.18)	0.91	(0.62–1.33)
More than 1 year	Ref		Ref	
Age				
18–24	1.13	(0.62–2.06)	0.96	(0.49–1.90)
25–29	0.77	(0.48–1.23)	0.78	(0.47–1.29)
30–34	0.73	(0.47–1.13)	0.72	(0.46–1.15)
35–39	Ref		Ref	
40–44	0.98	(0.66–1.45)	0.94	(0.62–1.41)
Marital status				
Single	1.28	(0.51–3.20)	1.06	(0.40–2.81)
Living together	2.10	(0.94–4.69)	1.76	(0.76–4.04)
Married	Ref		Ref	
Divorced/separated/widowed	1.39*	(1.01–1.92)	1.31	(0.93–1.85)
Number of living children				
0	1.71	(0.73–4.01)	2.00	(0.81–4.93)
1–2	1.06	(0.73–1.55)	1.04	(0.69–1.58)
3–4	Ref		Ref	
5+	0.93	(0.65–1.33)	0.80	(0.54–1.18)
Children living with HIV				
At least 1	0.81	(0.51–1.31)	0.74	(0.45–1.22)
None/don’t know	Ref		Ref	
Education				
None	1.14	(0.70–1.85)	1.09	(0.65–1.81)
Did not complete primary	Ref		Ref	
Completed primary	0.86	(0.52–1.43)	0.81	(0.48–1.38)
Secondary or more	3.07	(0.96–9.79)	2.91	(0.84–10.16)
Duration living in same village				
< 10 years	0.92	(0.67–1.27)	0.97	(0.68–1.39)
≥ 10 years	Ref		Ref	
Sub-County				
Butula	Ref		Ref	
Samia	1.92***	(1.33–2.77)	1.90***	(1.30–2.77)
Self-reported health status				
Poor/don’t know	1.65*	(1.02–2.66)	1.44	(0.87–2.38)
Fair	0.95	(0.69–1.31)	0.87	(0.62–1.21)
Good	Ref		Ref	

*p ≤ 0.05; **p ≤ 0.01; ***p ≤ 0.00

In the univariate models, women who experienced HIV-related discrimination and those who experienced violence were significantly more likely to have stronger feelings of internalized stigma (discrimination: OR 2.0; 95% CI 1.5–2.8; violence: OR 1.6; 5% CI 1.2–2.2). In the multivariate model while the association of experienced HIV-related discrimination and medium/high levels of internalized stigma remained significant (AOR: 1.8; 95% CI 1.3–2.6), the association between violence and internalized stigma was no longer significant.

Sub-county was the only other variable that showed a significant association with internalized stigma. Those who lived in Samia sub-county were nearly 2 times more likely (AOR 1.9; 95% CI 1.3–2.7) to have greater levels internalized stigma compared to women living in Butula sub-county. Covariates that were significant in the univariate model but were no longer significant in the multivariate model include marital status and self-reported health status.

## Discussion

This study showed that internalized stigma, experience of HIV-related discrimination and unintended pregnancy remain critical issues among WLHIV in south western Kenya. Just under half of the women agreed with at least one internalized stigma statement (47%) and 19% of women agreed with 3–7 statements (medium/high internalized stigma). Over half (56%) of the WLHIV surveyed said that their last pregnancy was not wanted at the time, consistent with other studies conducted in Kenya [6, 8, 13]. Many women also reported experiencing HIV-related discrimination (52%), and violence or abuse (41%) in the past 12 months. The experience of HIV-related discrimination within the past 12 months of the interview, and to some extent the experience of violence and abuse, also increased the likelihood of experiencing internalized stigma. While the effect of HIV-related stigma on health-seeking behaviors has been well documented [26–28], this study is one of the first to highlight the association between unintended pregnancy and internalized stigma.

WLHIV in this study were more likely to experience HIV-related internalized stigma if their last pregnancy was unintended compared to those whose last pregnancy was intended, despite experiences of discrimination or violence/abuse. Associations between internalized stigma and fertility intentions have been previously documented [42, 43]. Higher internalized stigma has been associated with higher fertility intentions in Uganda, possibly due to a desire to conform with normative behavior around childbirth, except in areas where childbearing among people living with HIV is stigmatized [42]. Similarly, personal and social pressure to be “normal” have led to people living with HIV to have children despite not wanting additional children [42]. Results from this and previous studies suggest that other factors in a woman’s life, beyond being HIV-positive, influence her perceptions and feelings towards herself. There appears to be a strong link between internalized stigma and a women’s fertility intentions, but the relationship may not be linear or casual. While the association between unintended pregnancy and internalized stigma remained significant when adjusting for experience of HIV-related discrimination and violence, further research is needed on whether experience of HIV-related discrimination or experience of violence mediates the association between unintended pregnancy and internalized stigma.

The timing of when a WLHIV learns of her seroconversion status (before versus during pregnancy) may influence whether a pregnancy is reported as wanted at the time or not [21] and her experience of internalized stigma [44]. While this study was unable to look at the timing of the pregnancy vis-a-vie HIV acquisition, research has shown that some WHLIV want to avoid pregnancy due to concerns of their children’s wellbeing [7, 8, 11] and therefore having an unintended pregnancy may manifested into internalized stigma, even years after pregnancy. Experiences of secondary stigma towards children have been documented and may also influences responses around pregnancy intentions [41].

More than half (57%) of respondents who reported that their last pregnancy was unintended were not using FP at the time of the pregnancy, similar to results from previous studies [4]. As many WLHIV prefer health facilities that provide specialized services for people living with HIV [8, 29, 30], these results suggest that WLHIV may not have the adequate access to reproductive health and FP information and services, including FP counseling for themselves and their partners, that meets their needs. A study in Kenya also found that women who were diagnosed with HIV during pregnancy and were connected to HIV care experienced lower levels of internalized stigma and depression [45]. Expanding accesses to integrated HIV/FP services or equipping health workers to provide unbiased reproductive health and FP services to WLHIV may help reduce barriers to contraception, help WLHIV plan for their families, and potentially reduce negative feelings towards themselves. In addition, some studies have found that women and couples desire to have additional children as they begin to feel healthier with ART [10, 12]. Therefore as WLHIV’s fertility intentions change over time, so do their contraceptive needs and healthcare providers should continue to regularly check in with WLHIV about those needs.

The measures of discrimination assessed in this study included a wide range of experiences and were found to be positively associated with internalized stigma. The results suggest that when a WLHIV experiences discrimination in different spheres of life, strong negative attitudes about herself can manifest. This is consistent with a study in Dominican Republic [46] that found WLHIV who reported depression and HIV-related stigma were more likely to report internalized stigma, and a study in the US which found that experienced stigma in the community had an indirect effect, through internalized stigma, on outcomes such as medication adherence and self-esteem [47], suggesting a correlation between the different levels of HIV-related stigma and discrimination. WLHIV in Samia were also more likely to experience medium/high internalized stigma compared to those in Butula. This could be because respondents from Samia sub-county are overrepresented in the analytical sample or because of other HIV projects that were being implemented in Butula sub-county at the time of the study.

While effective programs for reducing HIV-related stigma and discrimination have been well documented [48], less is known about strategies for reducing internalized stigma specifically [49]. Programs that have shown reductions in internalized stigma have addressed broader aspects in the lives of people living with HIV, such as social empowerment and economic strengthening, rather than internalized stigma directly [49]. As this study found a significant association between unintended pregnancy and internalized stigma, further research is needed to understand how reproductive health and FP programs that assist WHLIV with planning and spacing their pregnancies can also reduce internalized stigma for WLHIV. Since some WLHIV report concerns over the care and financial support of their families as reasons for not wanting additional children [7, 8, 11], income generation and empowerment activities targeted towards WHLIV could also be explored as ways to reduce feelings of internalized stigma in the event of an unintended pregnancy. Health systems interventions (e.g. reducing stockouts of and expanding access to ARTs) have also been shown to reduce internalized stigma [48]. Similar health systems interventions that aim to strengthen reproductive health and FP services and increase reproductive health and FP messaging through community health workers and existing support groups may also be beneficial in reducing internalized stigma among WLHIV.

### Limitations

Several limitations need to be considered in this study. One limitation to this analysis that should be considered is that respondents were not asked about when they learned of their seroconversion and therefore it is not known whether a woman’s last pregnancy occurred before or after she knew her HIV status. This can lead to changes in the intendedness of the pregnancy based on when she learned of her HIV status since pregnancy intention was asked at the time of the pregnancy but reported at the time of the interview, which is when she knows her HIV status. As pregnancy intention is asked at time of the interview, another limitation is that a woman may report her last pregnancy differently at the time of the interview (which is later) than at the time of the pregnancy. Other factors in a woman’s life, unrelated to HIV-status, such as martial or financial status, may influence this response at the time of the interview. Because the data were cross-sectional and unintended pregnancy was collected retrospectively, it is difficult to establish the directionality of the association and causality. Another limitation is that women enrolled in this study were all members of support groups for people living with HIV and the study did not interview WLHIV who did not belong to support groups. As result, study results pertain to a select group of women who are already motivated to seek services, and in theory should therefore have less internalized stigma.

## Conclusions

This study demonstrated that unintended pregnancy is associated with internalized stigma. FP and HIV programs, and others such as maternal and antenatal care services, should continue to explore how to best support WLHIV’s access to comprehensive reproductive health and FP services and continue to address HIV stigma and discrimination so that people living with HIV can access health services without fear of social or family rejection. While these results provide valuable insight for FP and HIV programming, additional research is needed on how unintended pregnancy and HIV-related discrimination influence internalized stigma among all WLHIV and whether participation in support groups mitigates experiencing internalized stigma.

## Supplementary Information

**Additional file 1.** The role of unintended pregnancy in internalized stigmaamong women living with HIV in Kenya.

## Data Availability

The dataset analyzed during the current study will be available at the USAID Development Data Library in 2021, https://www.usaid.gov/data. They will also be available from the corresponding author on reasonable request.

## Abbreviations

ART: Antiretroviral therapy
ARV: Antiretroviral
FP: Family planning
RH: Reproductive health
WLHIV: Women living with HIV
