# Supplementary material for: The role of unintended pregnancy in internalized stigma among women living with HIV in Kenya
Source: BMC Womens Health. 2021 Mar 17;21:106. doi: 10.1186/s12905-021-01224-5 (PMC7968281; doi:10.1186/s12905-021-01224-5)
Supplement: Supplementary file 1 — Additional file 1. The role of unintended pregnancy in internalized stigmaamong women living with HIV in Kenya. [file 12905_2021_1224_MOESM1_ESM.pdf]

The role of unintended pregnancy in internalized stigma among women living with HIV in Kenya

Sara A Chace Dwyer[1*§], Aparna Jain[1*], Wilson Liambila[2], Charlotte Warren[1]

1 Population Council, Washington D.C., USA

2 Population Council, Nairobi, Kenya

E-mail addresses of authors:

AJ: apjain@popcouncil.org

WL : wliambila@popcouncil.org

CW: cwarren@popcouncil.org

Questions used for the analysis in the manuscript "The role of unintended pregnancy in internalized stigma among women living with HIV in Kenya."

Suggested citation: Evidence Project. 2021. "Questions used for the analysis in the manuscript 'The role of unintended pregnancy in internalized stigma among women living with HIV in Kenya' in BMC Women's Health." Washington, D.C. & Nairobi, Kenya: Population Council, The Evidence Project.

**Ident.**	Sub county		______	
**B100**	How old are you now?<br>***Una umri wa miaka mingapi?***	Age (years)<br>Don't know	______<br>98	
**B102**	What is the highest level of schooling you attended? ***Umehitimu kiwango gani cha juu zaidi cha elimu?***	None/No education<br>Primary (no certificate/incomplete<br>Primacy (certificate/complete)<br>Secondary<br>Tertiary (college/university	00<br>01<br>02<br>03<br>04	
**B104**	What is your marital status currently?<br>***Hali yako ya ndoa kwa sasa ni ipi?***	Single and **not** in a regular relationship<br>Single with partner who lives elsewhere<br>Single and living with a partner<br>Married monogamous<br>Married polygamous<br>Divorced/separated<br>Widowed<br>Other (Specify)	01<br>02<br>03<br>04<br>05<br>06<br>07<br>88	
**B106**	How long have you been living continuously in this village? ***Umekuwa ukiishi hapa kwa muda gani?***<br>**[RECORD '00' IF LESS THAN ONE YEAR]**	Years<br>Always<br>Visitor	______<br>95<br>96	
**S300**	In general, would you say your health is...<br>***Kwa ujumla unawezasema afya yako ni...***<br>**[READ OUT RESPONSES & CIRCLE ONE]**	Very good (***Nzuri sana)***<br>Good (***Nzuri)***<br>Fair (***Kadri/wastani)***<br>Poor (***Mbaya)***<br>Don't know	01<br>02<br>03<br>04<br>98	
**F400**	Have you ever been pregnant?<br>***Umewahi kuwa mjamzito?***	Yes<br>No	01<br>00	 **S411**
**F402**	Are you currently pregnant?<br>***Uko mjamzito kwa sasa?***	Yes<br>No	01<br>00	 **S404**
**F404**	How many children have you given birth to in your lifetime?<br>***Umezaa watoto wangapi maishani mwako?***	Number of children<br>Pregnant with first child	______<br>96	**S408**
**F405**	How many of those children that you have given birth to in your lifetime are still alive? ***Wangapi kati ya hao watoto umewazaa maishani mwako wangali hai?***	Number of children<br>None	______<br>97	

**F406**	During the last **FIVE** years, how many children have you given birth to? ***Katika muda wa miaka mitano iliyopita, umezaa watoto wangapi?***	Number of children<br>None	____<br>97	**S411**
**F408**	At the time you became pregnant [with your last child], did you intend to become pregnant **then**, did you want to wait until **later**, or did you **not want** to have any (more) children? ***Wakati ulipopata mimba hii/mimba yako ya mwisho, ulitaka kupata mimba wakati huo; ulitaka kungoja hadi baadaye, au hukutaka mtoto yeyote/ watoto zaidi?*** **[READ OUT RESPONSES & CIRCLE ONE]**	Wanted to be pregnant then<br>Wanted to wait until later<br>Did not want any (more) children	01<br>02<br>03	
**S607**	Are any of your children/child HIV positive? ***Je, kuna yeyote kwa mtoto/watoto wako aliye na virusi vya UKIMWI?***	Not HIV positive<br>Some are/One is/ HIV positive<br>All are HIV positive<br>Don't know	01<br>02<br>03<br>99	
**S706**	Are you on antiretroviral (ARV) HIV treatment now? ***Unatumia ARV au matibabu ya virusi vya ukimwi kwa sasa?***	Yes<br>No	01<br>00	 **S708**
**S707**	When did you start taking antiretroviral HIV medication? ***Ulianza kutmia ARV lini?***	Less than 3 months ago<br>3–6 months ago<br>6months to one year ago<br>More than 1 year ago	01<br>02<br>03<br>04	
**S801**	In the past twelve (12) months, have you been excluded from family activities (*cooking, eating together, sleeping in the same room*) because you have HIV?	Yes<br>No<br>Don't know	1<br>0<br>99	
**S802**	In the past twelve (12) months, have you been aware of being gossiped about your HIV?	Yes<br>No<br>Don't know	1<br>0<br>99	
**S803**	In the past twelve (12) months, have you been rejected by a sexual partner because you have HIV?	Yes<br>No<br>Don't know	1<br>0<br>99	
**S804**	In the past twelve (12) months, have you ever been treated unfairly/or being discriminated against by a health care provider because of your HIV status?	Yes<br>No<br>Don't know	1<br>0<br>99	
**S806**	In the past twelve (12) months, have you been verbally insulted? (back biting, abusing, etc.)	Yes<br>No<br>Don't know	1<br>0<br>99	
**S807**	In the past twelve (12) months, have you been physically abused (kicked, punched, hit, slapped, shoved, etc.)?	Yes<br>No	1<br>0	
**S808**	In the past twelve (12) months, have you ever been sexually insulted or forced to have sex?	Yes<br>No	1<br>0	

**S810**	Having HIV makes me feel I'm a bad person.	Strongly disagree<br>Disagree<br>Agree<br>Strongly Agree<br>Don't know	1<br>2<br>3<br>4<br>98	
**S811**	*I feel I'm not as good as others because I have HIV.*	Strongly disagree<br>Disagree<br>Agree<br>Strongly Agree<br>Don't know	1<br>2<br>3<br>4<br>98	
**S812**	Having HIV makes me feel unclean.	Strongly disagree<br>Disagree<br>Agree<br>Strongly Agree<br>Don't know	1<br>2<br>3<br>4<br>98	
**S813**	Having HIV in my body is disgusting to me.	Strongly disagree<br>Disagree<br>Agree<br>Strongly Agree<br>Don't know	1<br>2<br>3<br>4<br>98	
**S814**	People's attitudes towards people living with HIV make me feel worse about myself.	Strongly disagree<br>Disagree<br>Agree<br>Strongly Agree<br>Don't know	1<br>2<br>3<br>4<br>98	
**S815**	I feel guilty because I have HIV.	Strongly disagree<br>Disagree<br>Agree<br>Strongly Agree<br>Don't know	1<br>2<br>3<br>4<br>98	
**S816**	I feel ashamed of having HIV.	Strongly disagree<br>Disagree<br>Agree<br>Strongly Agree<br>Don't know	1<br>2<br>3<br>4<br>98	
